# Brain morphometry in Pontocerebellar Hypoplasia type 2

**DOI:** 10.1186/s13023-016-0481-4

**Published:** 2016-07-19

**Authors:** Kaspar Ekert, Samuel Groeschel, Iciar Sánchez-Albisua, Saskia Frölich, Andrea Dieckmann, Corinna Engel, Ingeborg Krägeloh-Mann

**Affiliations:** Department of Child Neurology, Children’s Hospital, University of Tübingen, Hoppe-Seyler-Str. 1, 72072 Tübingen, Germany; Department of Neuropediatrics, Jena University Hospital, Bachstraße 18, 07743 Jena, Germany

**Keywords:** Pontocerebellar hypoplasia type 2, Mental retardation, Microcephaly, Brain morphometry, Cerebellum, Brain stem, Pons, Frontal lobe

## Abstract

**Background:**

Pontocerebellar hypoplasia type 2 (PCH2) is caused by a defect in the *TSEN54*-gene and leads to severe and early disruption of brain development, especially of cerebellum and pons. The aim of this work was to quantify the infra- and supratentorial brain growth during postnatal brain development in children with PCH2.

**Methods:**

MRI data of 24 children with PCH2 (age 0.02–17 years., 13 females) were analysed volumetrically and compared to images of 24 typically developing age- and gender-matched children. All children with PCH2 had the homozygous p.A307S mutation in the *TSEN54*-gene. In 5 patients follow-up MRI investigations were available.

Images of the children with PCH2 were available either on film (*n* = 12) or in digital format (*n* = 21). Images on film were digitalized. Brain structures were manually masked and further adjusted semi-automatically using intensity thresholding to exclude CSF. Volumes of cerebellum, brain stem, and pons were measured, as well as supratentorial brain and frontal lobe volume. For validation of the method part of the digital images were processed as images on film. In addition, intra- and inter-rater variabilities were tested.

**Results:**

Children with PCH2 showed reduced volumes of all measured brain structures compared to healthy controls. Severely hypoplastic cerebellum, pons and brain stem only slightly increased in size postnatally. Supratentorial brain volume also showed reduced growth compared to the healthy controls. Differences between patients and controls could already be seen at birth but became more significant during childhood. Validation of the method showed high precision and reproducibility.

**Conclusions:**

In a genetically very homogenous group of children with PCH2 severely hypoplastic infratentorial structures, the hallmark of the disease, showed only slight increase in volume postnatally. Supratentorial brain structures, which are considered normal at birth, also showed smaller volumes neonatally and a lower growth rate compared to controls, leading to severe microcephaly. Volume loss, however, could not be observed during the first years of life. This argues for a severe disruption of the cerebellar-cerebral networks during pre- and postnatal development caused by a primary cerebellar dysfunction, rather than postnatal neurodegeneration. The developmental progress in these children, although little, further supports this.

**Electronic supplementary material:**

The online version of this article (doi:10.1186/s13023-016-0481-4) contains supplementary material, which is available to authorized users.

## Background

Pontocerebellar hypoplasias (PCH) are a group of very rare miscellaneous disorders of prenatal onset characterized by an abnormally small cerebellum and ventral pons. The estimated incidence is lower than 1:200.000 [1, 2]. The main clinical feature is severe psychomotor retardation. In many cases, the condition is fatal early in life. To date, seven types of non-syndromic PCH have been defined on the basis of clinical and genetic criteria [1, 3]. PCH Type 2 (PCH2) is the most common form and shows autosomal recessive inheritance. Ninety percent of PCH2 cases carry a missense mutation (p.A307S) in the *TSEN54*-gene on chromosome 17, which defines the PCH2A form (OMIM* 608755) [2, 3]. Other PCH2 types (PCH2B, PCH2C and PCH2D) are caused by mutations in different genes such as *TSEN2*, *TSEN34* and *SEPSECS* respectively.

From a neuropathological point of view (according to a study in the pregenetic era), PCH2 has been considered a neurodegenerative disorder, but both hypoplastic (short cerebellar folia with poor branching) and degenerative changes have been reported [4]. Most degenerative signs (ie sharply demarcated areas where cerebellar cortex lost its full thickness) are thought to result from regression at an early stage of development. Prenatal imaging findings reporting normal cerebellar structures in the second trimester of pregnancy also argue in this respect [5, 6]. But there is some evidence for later neurodegenerative changes from a report in one patient who died at 22 years where cystic cerebellar degeneration and vascular changes limited to the cerebellum were described, such as intimal proliferation and splitting of the elastica interna [4].

From a clinical point of view, neurodevelopment in an early onset neurodegenerative disorder is expected to be considerably limited and cognitive and voluntary motor development in PCH2A have been referred to as absent [1, 3]. However, we could recently show in a larger cohort of patients with PCH2A that even the most affected patients made some progress, although on a small scale [6]. Only around 10 % of patients lost some functions they had achieved earlier [6]. This is supported by single case reports on some neurodevelopmental progress in affected individuals [3].

A main feature of PCH2A is severe microcephaly during development. At birth most affected children are normocephalic and supratentorial brain imaging is-in contrast to cerebellum and pons-reported as unremarkable [3]. The postnatal progressive microcephaly may have two explanations: 1) the cerebellum establishes millions of projections to the telencephalon, especially to the frontal lobe [7–9] during foetal and early infantile life. In PCH2 this early development might be disturbed due to the severe cerebellar hypoplasia. In this case, a reduced growth of the telencephalon, especially the frontal lobes, would be expected; 2) on the other hand, an ongoing neurodegeneration might also play a role. Evidence of telencephalic volume loss indicating brain atrophy at later ages would support this hypothesis.

Volumetric MRI analysis allows the in-vivo quantification of brain development. The aim of this study was to analyse intracranial volumes in a genetically homogeneous group of children with PCH2A, eg all carrying the missense mutation (p.A307S) in the *TSEN54*-gene. This could provide some insight into the impact of the gene defect involved and its protein product in relation to brain function and development.

## Methods

### Subjects and MRI

All children with PCH2 were recruited as part of a natural history study [6] and with the support of the PCH2 parents’ organization in Germany and Switzerland. Subjects were included if they met the clinical and MRI-based diagnosis accompanied by molecular testing of PCH 2 (homozygous mutation c.919G > T, p.A307S in *TSEN54*-gene).

Thirty six MRI images from 27 children with PCH2 could be collected either on film or as digital copy in DICOM format. Four MRI scans had to be excluded for one of the following reasons: Either the MRI films did not contain a measurable scale (*n* = 3) or the sequence did not cover the entire brain for volumetric analysis (*n* = 1). 32 MR images of 24 children with PCH2 remained for further analysis. Gender was almost evenly distributed with 11 males and 13 females. The age range was 0.02 to 17.06 years with a median of 0.81 years (95 % confidence interval 0.6969–4.1286). It is also worthy of note that the head circumference at birth was normal in 84 % of these infants. 13 MRI scans of 5 children were available for longitudinal analysis with an age range at MRI of 0.02 to 10.5 years and a median of 0.53 years.

Conventional MRI of children with PCH2 consisted of T1-, T1-IR, T2-, and FLAIR-weighted sequences acquired in different planes (see Table 1 for details) with high in-plane resolution and a mean slice thickness of 4.41 mm (±1.94). Four children had high-resolution T1-weighted images with a voxel size of 1x1x1mm. As a control group, MRI data of age- and gender-matched typically developing children was obtained from the NIH Paediatric Data Repository [10]. For each MRI scan of the children with PCH2, a gender- and age-matched control scan was chosen, resulting in 13 females and 11 males with the following age distribution: median 0.79 years (95 % confidence interval 0.7006–3.9327, range 0.02–16.08 years). Image sequences consisted of high-resolution T1-weighted sequences with a mean voxel size of 1.01 (±0.01) x 0.99 (±0.01) × 2.82 mm (±0.10).

**Table 1:** MRI sequence details for children with PCH2

Plane		T1	T2	FLAIR	T1-IR	Total
Axial	Number	13	3	0	6	22
	Slice thickness mean (SD)	5.25 mm (±1.67 mm)				
Coronal	Number	8	8	2	2	20
	Slice thickness mean (SD)	4.36 mm (±2.01 mm)				
Sagittal	Number	11	6	0	0	17
	Slice thickness mean (SD)	3.47 mm (±1.68 mm)				

### MRI analysis

**Fig. 1:**
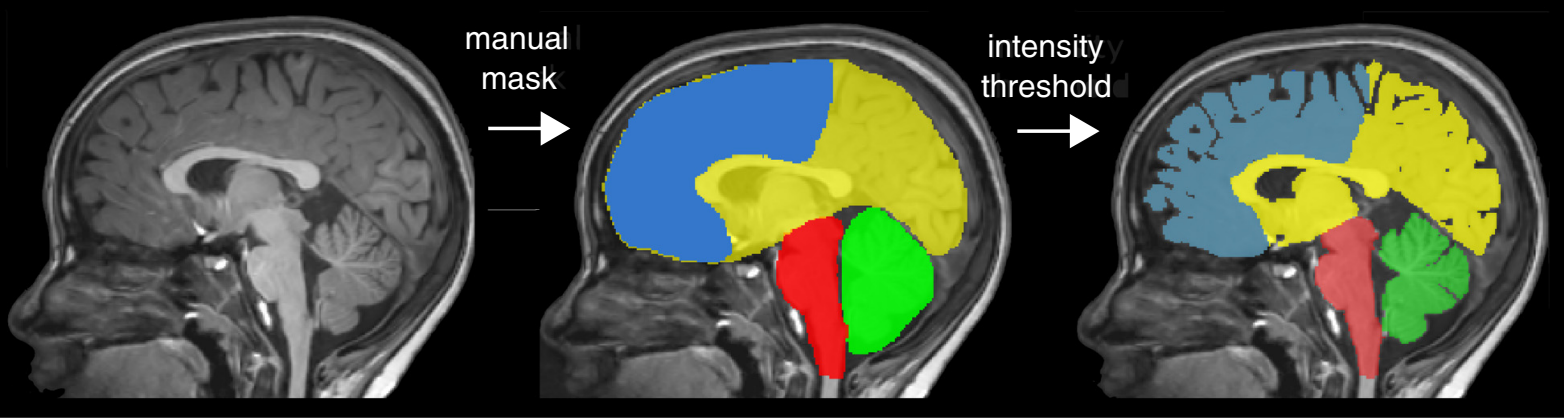
Outline of methodological approach. In the first step, rough brain masks were manually created for cerebellum, brain stem, supratentorial brain volume and frontal lobe. Following this, image intensity threshold was automatically applied and manually adjusted, in order to remove CSF signal within the brain mask

MRI both on film and in digital (DICOM) format were analysed using the same two-step approach as illustrated in Fig. 1. First, a manual (rough) mask of the desired brain structure was created. The mask was then further refined by setting an intensity threshold in order to remove CSF signal resulting in the volume of the particular brain structure.

Images on film were converted into tiff-format by using a high resolution camera mounted over the MRI film. A picture was taken of the image slice under constant conditions, in particular image resolution and lighting were maintained. The tiff-files were further analysed using ImageJ [11], adding all slices into one file, creating the mask of the brain structure (Fig. 1), applying an intensity threshold and using the metric scale on the films to calculate the area of the structure in each slice. The area was then multiplied by the slice thickness. Slice gaps were added to the slice thickness indicated on the film.

Images in digital (DICOM) format were converted into nifti-format using MRtrix [12]. Rough manual masks (Fig. 1) of the brain structures were created using ITKSnap [13]. The mask was then further refined by applying a manual threshold reducing the CSF signal intensity from the masked structure using MRtrix and finally the number of voxels was multiplied by the voxel size resulting in the volume of the particular structure.

### Definition of brain structures

All available sequence planes were used for orientation and, when available, the high-resolution T1-weighted image was used for the volumetric analysis. The cerebellar volume was measured in coronal view, the cerebellar peduncles were included. Supratentorial brain volume was measured in axial plane on T1-weighted images, when available in good quality; otherwise other (axial) sequences were used. The cerebellar tentorium was defined as the caudal border as well as the caudal end of the thalamus, including the thalamus into the segmentation of the supratentorial brain volume. Brain stem volume was measured in sagittal view. The cranial boundary was drawn below the third ventricle. The cerebellar peduncles were not included. The caudal boundary was defined by the foramen magnum as previously described [14]. The pons in patients with PCH2 is extremely small in relation to the partly large slice thickness of the images causing partial volume effects; therefore the area of the pons (not its volume) was measured in the axial plane at the level of the middle cerebellar peduncles.

### Statistical analysis

For examination of the dependence between volume of different brain structures (cerebellum, pons area, brain stem, supratentorial brain and frontal lobe in ml) and age (in years) regression analyses have been performed. After considering different equations, a model allowing for different intercept and slope, including the age as its natural logarithm, revealed the best fit of the data (y = a + b*lnx). Due to the fact that for only a few children more than one image over the course of their disease was available, the most recent image available was included (ie 24 images of 24 children).

Furthermore, longitudinal measurements of children with PCH2 were plotted over their age (13 images of 5 children) and analysed visually in order to test whether the results from the cross-sectional growth curves could be reproduced by the longitudinal examples.

### Validation

For validation of the two different image preparation approaches, part of the digital images (randomly selected, in nifti format, *n* = 10) were converted into pixel-images in tiff-format (using screenshots) and measured using the ImageJ software (by KE). A metric scale was added to the images by creating a line of a certain number of voxels. Furthermore, intra- (KE) and inter-rater (SG, KE) tests were done in randomly selected subjects (*n* = 10) using the intra-class correlation coefficient (ICC) for volume agreement, and the dice coefficient for spatial overlap [15]. This was done both for the cerebellum and for the supratentorial brain volume in order to test the precision of the method in both a small and large brain structure.

## Results

### Cerebellar volume

**Fig. 2:**
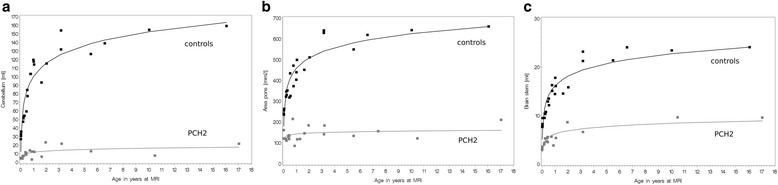
Scatterplots showing **a** the cerebellar volumes, **b** the pons area, and **c** the volume of the brain stem of children with PCH2 (in *grey*) compared to typically developing children (in *black*) over the age at MRI. Note that children with PCH2 show an increase in their infratentorial structures during their first 1 or 2 years

As can be seen in Fig. 2a (Additional file 1: Figure S1), cerebellar volume was already clearly smaller at birth in children with PCH2 than in controls (11.6 ml vs. 99.7 ml, respectively, *p* < 0.0001) with hardly any increase in volume (2.1 ml vs. 22.9 ml per year using the logarithm of age, respectively, *p* < 0.0001). After age 2, no further relevant cerebellar growth was seen in children with PCH2, whereas in controls cerebellar volume increased clearly within the first ten years of life.

### Brain stem and pons

Brain stem volume and pons area showed a similar pattern. As can be seen in Fig. 2b, c, brain stem volume and pons area were already smaller at birth in children with PCH2 than in controls (143 vs. 457 ml for pons area, and 6.2 vs. 16.1 ml for brain stem) and showed little growth over the observed age range (6.2 vs. 72.7 ml for pons area, and 1 vs. 2.9 ml for brain stem). All differences revealed p-values <0.0001. After the age of 1 or 2 years, no further relevant growth of brain stem and pons was seen in children with PCH2, this was especially evident in the pons area, whereas in controls brain stem and pons increased clearly within the first ten years of life.

### Supratentorial brain volume

**Fig. 3:**
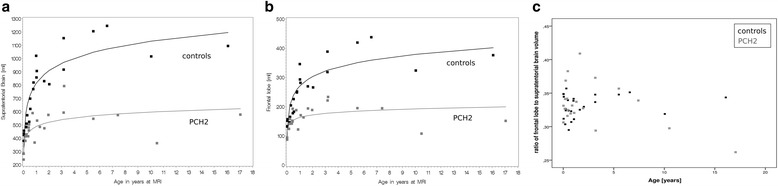
Scatterplots showing **a** the supratentorial brain volumes, and **b** the volume of the frontal lobes of children with PCH2 (in *grey*) compared to typically developing children (in *black*) over the age at MRI. Note that children with PCH2 show an increase in volume of their supratentorial structures during their first years of life. **c** The ratio of frontal **a** to supratentorial volumes **b** is shown over the age range

In addition, supratentorial brain volume at birth was smaller in children with PCH2 than in controls (488 vs. 813 ml, *p* < 0.0001, Fig. 3a) albeit to a much lower extent than what was seen in cerebellar volume. Brain volume increase over age was also lower in patients than in controls (48 vs. 138 ml, *p* < 0.0001). Brain volume increased in children with PCH2 until around 3 or 4 years of age, with no clear changes thereafter, whereas in controls brain volume increased throughout the first ten years of life.

### Frontal lobe volume

Whilst analysing the volume of the frontal lobe of the brain separately, the same trend was observed. Volumes of frontal lobes were already reduced at birth compared to the controls (163 vs. 270 ml, *p* < 0.0001). And the subsequent growth of the frontal lobe was found to be reduced in children with PCH2 (13 vs. 47 ml, *p* < 0.0001), resulting in an increasing difference of frontal lobe volumes between the two groups. The ratio of the frontal lobe to the whole supratentorial brain volume is shown in Fig. 3c. There was no overall difference in this ratio between the two groups. However, the children with PCH2 over the age of 10 years showed a lower ratio of frontal lobe to supratentorial brain volumes compared to their age- and gender matched controls.

### Validation

The resulting volumes of the two-image preparation approaches (on film and digital images), were compared. The volume agreement (using the ICC) was 0.998 (95 %-confidence interval [CI] 0.992–0.999) for the supratentorial brain volumes and 0.998 (CI 0.990–0.999) for the cerebellar volumes.

The intra-rater variability test showed a volume agreement (ICC) for the supratentorial brain volume of 0.999 (CI 0.996–1.0) and for the cerebellar volume of 0.998 (CI 0.993–1.0). The spatial overlap between two measurements by one rater was also very high with a mean dice coefficient of 0.97 (±0.018) for the supratentorial brain and 0.90 (±0.058) for the cerebellum.

The inter-rater variability test resulted in a volume agreement (ICC) for the supratentorial brain volume of 0.999 (0.996–1.0) and for the cerebellar volume of 0.998 (CI 0.991–1.0). The spatial overlap between the two raters was also very high with a mean dice coefficient of 0.98 (±0.011) for the supratentorial brain and 0.94 (±0.033) for the cerebellum.

### Longitudinal analysis

**Fig. 4:**
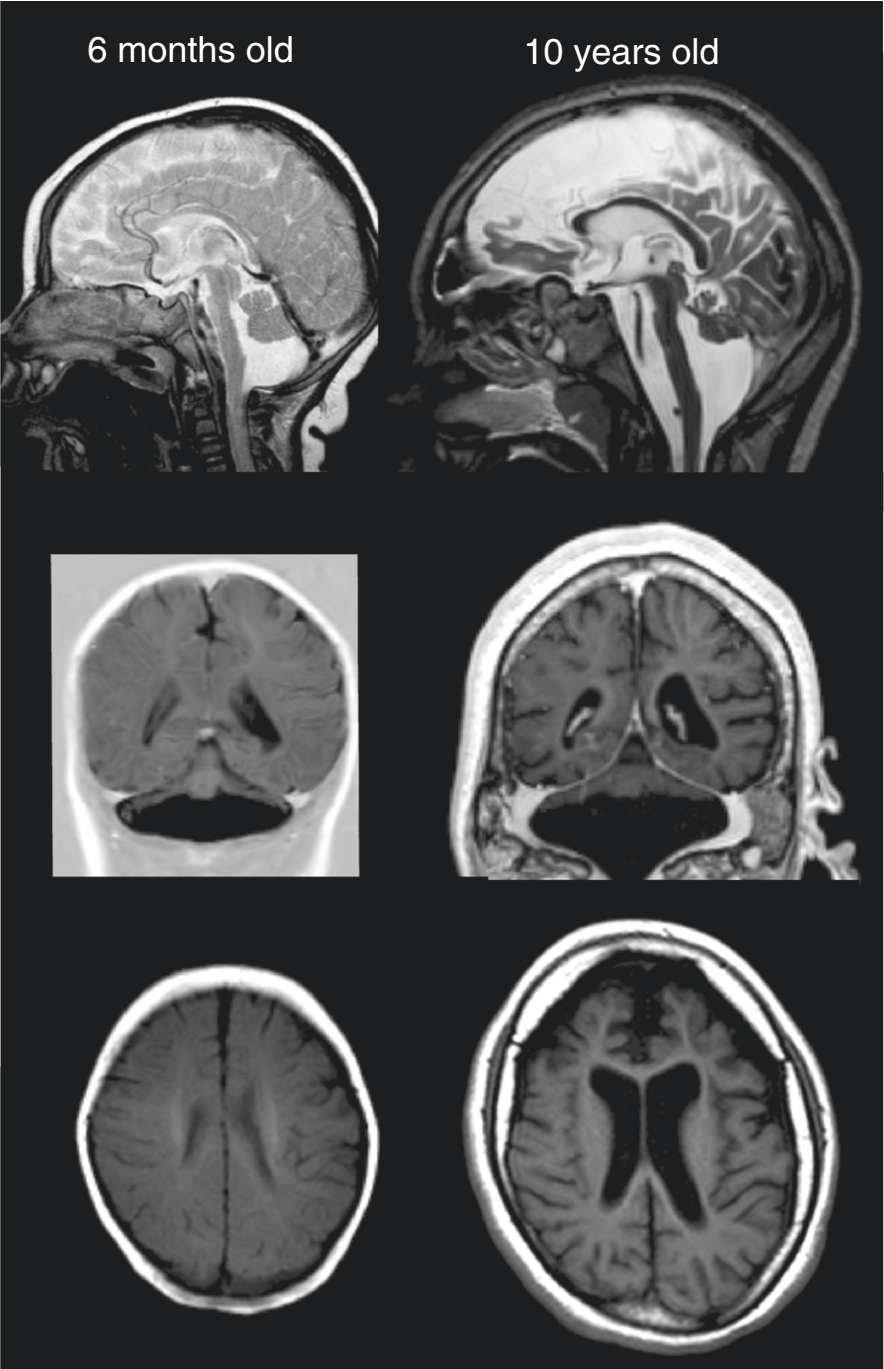
Example longitudinal MRI examination in one girl with PCH2A at the age of 6 months and 10 years, illustrating the severe hypoplasia of pons and cerebellum already in infancy. Note that the scale of both images is identical, indicating hardly any brain volume increase after ten years of life (but thickened skull), with larger CSF spaces suggesting atrophy, with some frontal lobe dominance

Only a few patients (*n* = 5) had follow-up MRI examinations. The visual analysis of these images confirmed the trends observed in the cross-sectional analysis. All showed some increase in volume during the first 1–2 years. Only one patient with PCH2 had a long-term follow-up MRI; at 6 months and 10 years of age. Figure 4 demonstrates the longitudinal changes over time. Volumetrically, pons, cerebellum and supratentorial brain structures were slightly smaller on follow-up, and on visual inspection there was pronounced enlargement of CSF spaces suggesting atrophy (Additional file 2: Figure S2).

## Discussion

For the first time, we investigated the growth of several brain structures in pontocerebellar hypoplasia over the first 17 years of life. This study represents volumetric data of a genetically very homogeneous group of children. All children had the homozygous p.A307S mutation in the *TSEN54*-gene. In a previous study on the natural course of this condition [6], we showed that affected patients all had a severe clinical phenotype and reduced life expectancy. Nevertheless, all children made some progress in both cognitive and motor domains, but on a low level; only 10 % lost achieved abilities upon follow-up.

A main clinical feature was progressive microcephaly during development. Most but not all affected children were normocephalic at birth (within 2 standard deviations), as also reported by others [3], but all showed progressive microcephaly falling below 5 standard deviations from the mean already in the second year of life, whereas both failure to thrive and short stature only fell below 2 standard deviations [6].

On conventional MRI, infratentorial structures are reported clearly abnormal shortly after birth and are the basis - along with clinical criteria - for defining PCH2: hypoplasia of the pons and dragonfly-like atrophy of cerebellar hemispheres with relative sparing of the flocculus and vermis [3]. In contrast, supratentorial structures are referred to as unremarkable at birth.

Using volumetric measures, we were able to quantify the extreme hypoplasia of pons, brain stem and cerebellum at birth in comparison to controls, and also show that supratentorial brain volume was clearly smaller in children with PCH2 already at birth compared to typically developing children. A selection bias for MRI indication is unlikely, since patients with PCH2 are clinically severely affected already in the first days of life and the MRI is performed due to the clinical abnormalities, and not as a result of a possible microcephaly. In fact, 84 % of the examined patients in our study were normocephalic at birth.

Infratentorial brain structures showed some growth only in infancy which was, however, at a clearly slower growth rate compared to typically developing children. Later in childhood volumes of infratentorial structures did not increase further compared to the volume increase in typically developing children. MRI studies of normal brain development clearly show increases of infratentorial structures not only perinatally [16] but also during the first decade of life [17, 18].

The growth rate of supratentorial brain structures was also reduced compared to typically developing children. Volume increase still could be observed during the first three to four years of life, this was seen when analysing cross-sectional as well as longitudinal data. However, after the age of around 4 or 5 years, cross-sectional data did not show further relevant increase and even suggest some degree of volume decrease. Observation of a single case with a follow-up over almost ten years supported this by giving evidence for atrophy, as CSF spaces were markedly enlarged on follow-up (Fig. 4) and volumes were slightly smaller. This has to be interpreted with caution, however, as our data during the second decade of life are very sparse, the inter-subject variability is high and infra- and supratentorial brain volumes decrease mildly during normal development in the second decade of life [18, 19].

By measuring brain structures volumetrically in PCH2 during development we hoped to gain some insight into the pathomechanisms induced by the gene defect. We considered two hypotheses:

1. The splicing disorder interferes with the growth of pons and cerebellum during foetal and early infantile life, probably due to early neurodegeneration. Supratentorial brain growth would be secondarily affected, due to the fact that projections to the telencephalon cannot or only sparsely develop. As these projections are especially abundant in the frontal lobe during typical development, growth of the frontal lobe would be predominantly affected during the early postnatal development. Our data show smaller brain volumes already at birth not only for infra- but to some extent also for supratentorial structures. Growth curves for both were slower compared to those of typically developing children. However, during the first years of life a clear increase in supratentorial brain volumes was evident, and also of infratentorial structures to some degree. This seems to support the hypothesis of primary early disruption of cerebellar growth with a secondary impact on supratentorial development. We could not confirm, however, that the frontal lobe is selectively affected in volume. Instead we found that both the growth of the frontal lobe as well as that of the whole supratentorial brain volume were equally affected during the first years of life. Given the large amount of cerebellar neurons [20] and high structural and functional connectivity to the whole cerebral cortex not only to the frontal lobe [9, 21, 22], it is not surprising that the severe hypoplasia of the cerebellum severely affects the development of cerebellar-cerebral projections, which leads to severe global disturbance of the supratentorial brain development, resulting in a severe generalised developmental dysfunction of the affected children [6].
  Nevertheless, in one longer longitudinal observation over nearly ten years, there was a predominant involvement of the frontal lobe in relation to the rest of the supratentorial brain volume. However, this finding might be better explained as an effect of later neurodegeneration, adding to the effect of early secondary disruption of supratentorial brain development.
2. The second hypothesis was that of an ongoing neurodegeneration, as suggested before [4], which would lead to loss of brain volume or atrophy on follow-up. However, we could not see any atrophy or volume loss during the first years of life. Instead found volume increases, also in our longitudinal cases. In a few older children, however, and in one longitudinal observation we found some evidence for volume loss. This might indicate that neurodegeneration of supratentorial structures is relevant at least during later stages of the disease. However, this has to be interpreted with caution, as our data in the later stages were sparse. Extremely low birth weight infants may have exclusive cerebellar injury resembling MRI aspects of PCH [23]. Their clinical outcome is mainly characterized by considerable cognitive delay and also neuromotor impairment, but not to such an extent as characteristic in PCH2A patients. This would also argue for additional pathomechanisms than disrupted cerebellar-cerebral loops.

## Conclusion

In conclusion, in PCH2A the volumes of pons, cerebellum and brainstem were greatly reduced at birth, which is the hallmark of the disease, showing some further growth only in infancy. Interestingly, also the supratentorial brain structures were clearly reduced already at birth, although the children were mostly normocephalic. The supratentorial growth rate during the first 3–4 years of life was much slower than in typically developing children and was not observed thereafter. Thus, our data rather support the hypothesis of a severe disruption of the cerebellar-cerebral networks caused by a primary cerebellar dysfunction with some ongoing increase in infra- and supratentorial brain volumes after birth. This is also supported by our clinical data which show some developmental progress in all children. However, in our few older subjects we also found evidence of volume loss, suggesting some additional effect of neurodegeneration at later stages of the disease.

### Methodological limitations

A limitation of the study is the heterogeneous MRI data, which originated from different MR scanners and sequences. Furthermore, for a volumetric study, high-resolution MRI data would have been ideal to reduce partial volume effects. However, we were able to show that the precision and reproducibility of our methodology yielded excellent results with high intra- and inter-rater reliabilities. It has to be kept in mind that we were not able to assess the accuracy of our method in the absence of a ground truth. Nevertheless, with our methodological approach we were able to make use of extremely rare MRI data and perform volumetric analyses with both images on film and in digital format with high reproducibility.

Another limitation of the study is that, due to clinical reasons, most MRIs were performed on younger patients and only few of them on older patients. Although the findings in the young age group remain strong, this increases the uncertainty of the curve estimation in the older age group. Furthermore, as previously discussed [19] longitudinal data would be ideal in studying brain development. Making inferences about the growth of brain structures over time from our cross-sectional data has to be done carefully. However, some longitudinal observations in our data (Fig. 4), although sparse, seem to confirm the trend observed in the cross-sectional analysis. Moreover, it would otherwise not be possible to report data on a disease with an incidence lower than 1:200.000, where longitudinal measurements are ethically difficult to justify.

## Abbreviations

CSF, cerebrospinal fluid; DICOM, digital imaging and communications in medicine; ICC, intraclass correlation coefficient; MRI, magnetic resonance imaging; PCH2, pontocerebellar hypoplasia type 2; SD, standard deviation

## Additional files

Additional file 1: Figure S1. Extracts of scatterplots of figure 2 and 3 of the manuscript over only the first 4 years of life in order to illustrate the early postnatal increase of all brain structures, with the infratentorial structures (A–C) growing to a slower degree compared to supratentorial volumes (D–E). Note that the frontal lobe is not predominantly affected. (PDF 94 kb) Additional file 2: Figure S2. Longitudinal data demonstrating increase in volumes over the first years of life and some volume decrease in one long-term follow-up over 10 years. (PDF 125 kb)
