# Supplementary material for: Brain morphometry in Pontocerebellar Hypoplasia type 2
Source: Orphanet J Rare Dis. 2016 Jul 19;11:100. doi: 10.1186/s13023-016-0481-4 (PMC4950429; doi:10.1186/s13023-016-0481-4)
Supplement: Additional file 1: Figure S1. — Extracts of scatterplots of figure 2 and 3 of the manuscript over only the first 4 years of life in order to illustrate the early postnatal increase of all brain structures, with the infratentorial structures (A–C) growing to a slower degree compared to supratentorial volumes (D–E). Note that the frontal lobe is not predominantly affected. (PDF 94 kb) [file 13023_2016_481_MOESM1_ESM.pdf]

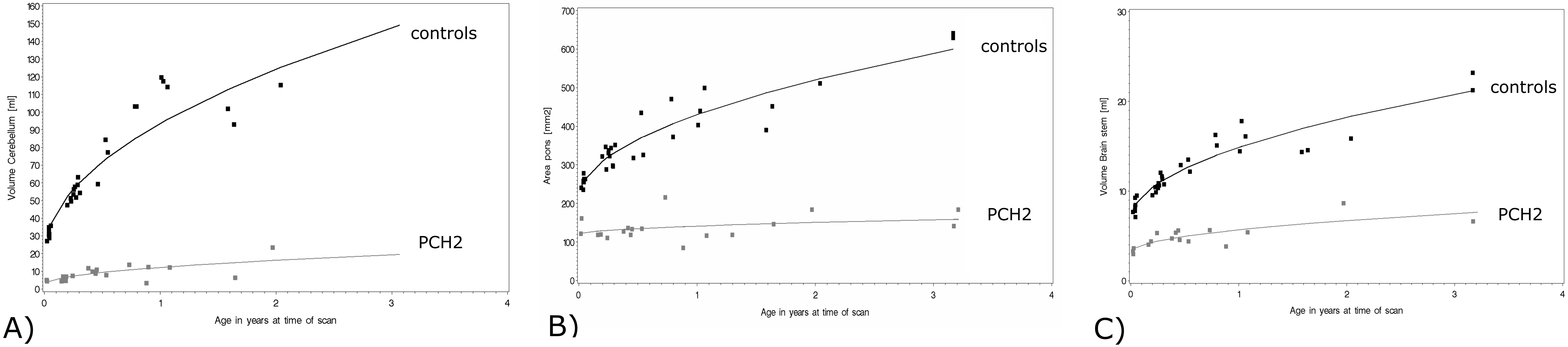

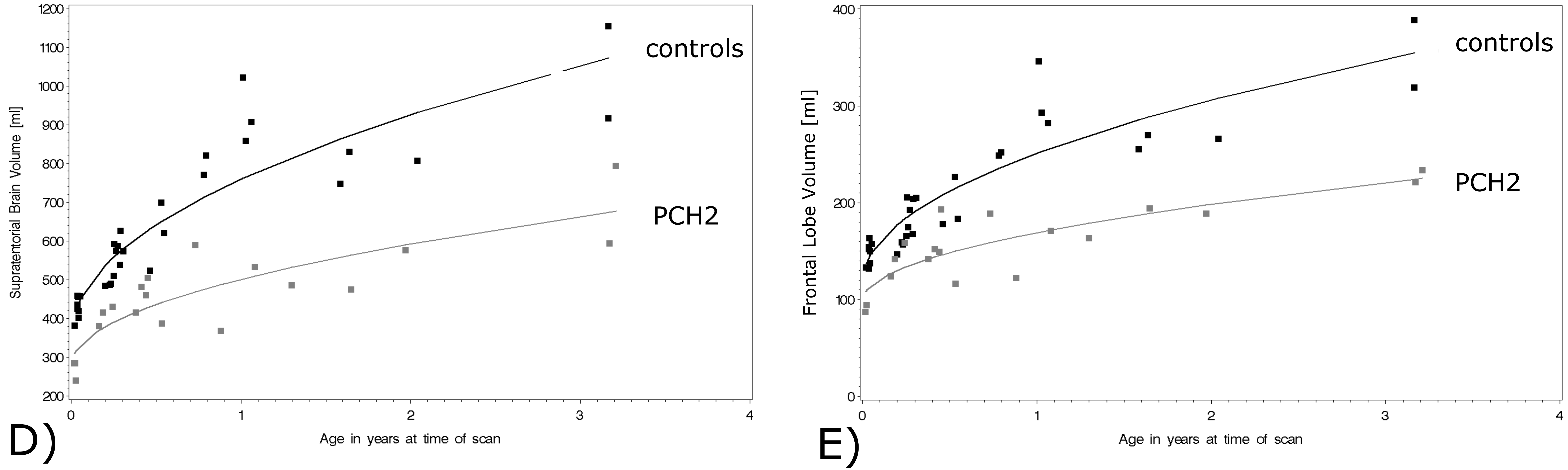

Online material figure 1:

Extracts of scatterplots of Figure 2 and 3 of the manuscript over only the first 4 years of life in order to illustrate the early postnatal increase of all brain structures, with the infratentorial structures (A-C) growing to a slower degree compared to supratentorial volumes (D-E). Note, that the frontal lobe is not predominantly affected.
