# Supplementary material for: Brain morphometry in Pontocerebellar Hypoplasia type 2
Source: Orphanet J Rare Dis. 2016 Jul 19;11:100. doi: 10.1186/s13023-016-0481-4 (PMC4950429; doi:10.1186/s13023-016-0481-4)
Supplement: Additional file 2: Figure S2. — Longitudinal data demonstrating increase in volumes over the first years of life and some volume decrease in one long-term follow-up over 10 years. (PDF 125 kb) [file 13023_2016_481_MOESM2_ESM.pdf]

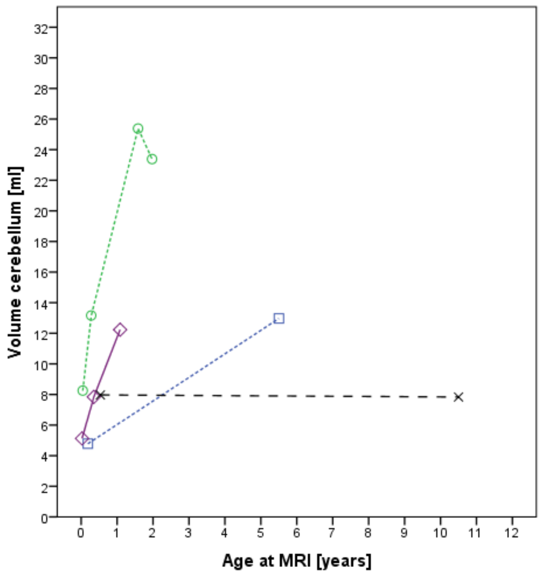

A)

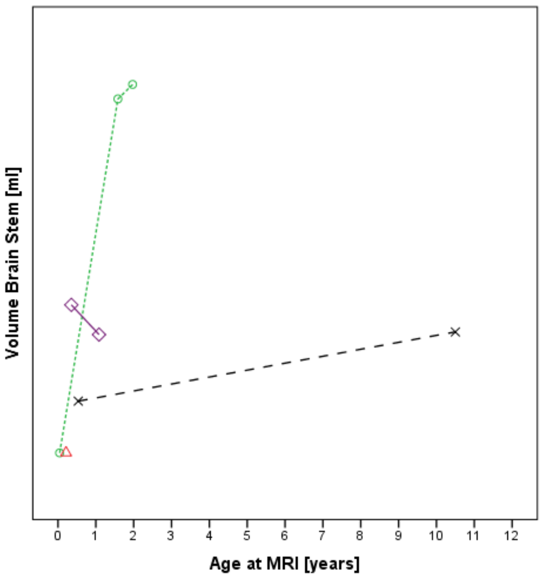

B)

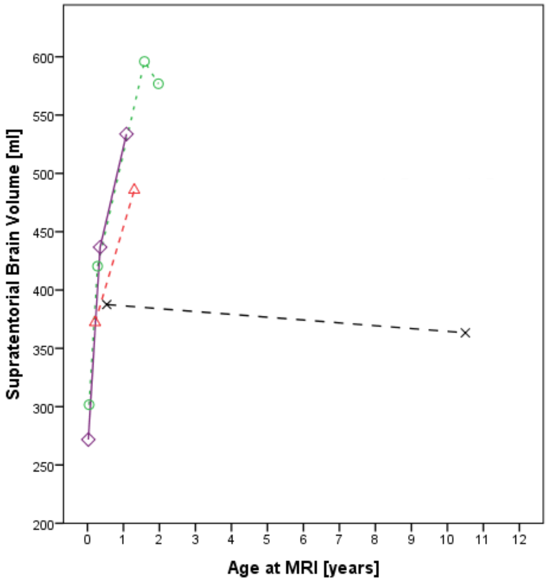

C)

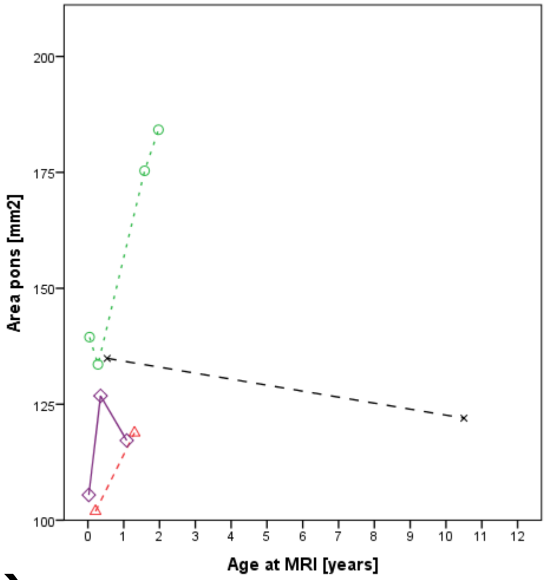

D)

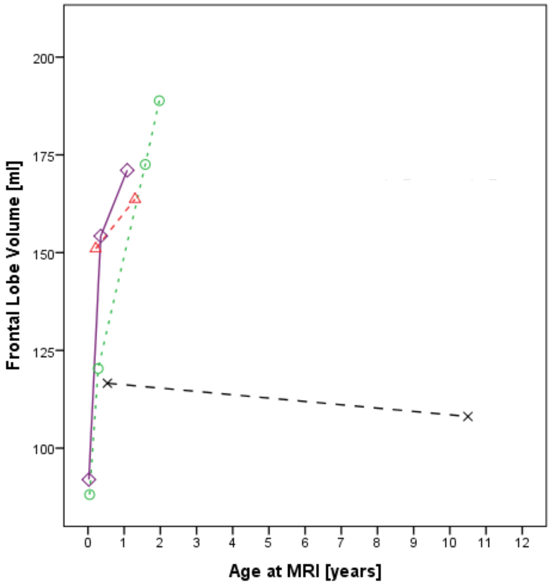

E)

Online figure 2:

Longitudinal data demonstrating increase in volumes over the first years of life and partly volume decrease in one long-term follow-up over 10 years.
